# The Architecture of Contemporary Care Networks for Rare Movement Disorders: Leveraging the ParkinsonNet Experience

**DOI:** 10.3389/fneur.2021.638853

**Published:** 2021-03-30

**Authors:** Bart P. van de Warrenburg, Mark Tiemessen, Marten Munneke, Bastiaan R. Bloem

**Affiliations:** Department of Neurology, Radboud University Medical Centre, Centre of Expertise for Parkinson and Movement Disorders, Donders Institute for Brain, Cognition and Behaviour, Nijmegen, Netherlands

**Keywords:** network, Parkinson's disease, rare disorders, integrated care, telemedicine, movement disorder

## Abstract

In this paper, we present a universal model for implementing network care for persons living with chronic diseases, specifically those with rare movement disorders. Building on our longstanding experience with ParkinsonNet, an integrated care network for persons living with Parkinson's disease or a form of atypical parkinsonism, we provide a series of generic, supportive building blocks to (re)design comparable care networks. We discuss the specific challenges related to rare movement disorders and how these challenges can inform a tailored implementation strategy, using the basic building blocks to offer practical guidance. Lastly, we identify three main priorities to facilitate network development for these rare diseases. These include the clustering of different types of rare movement disorders at the network level, the implementation of supportive technology, and the development of interdisciplinary guidelines.

## Introduction

Over the past decade, we have witnessed the emergence of care networks for a wide variety of diseases. Driving forces have been partially externally driven (e.g., governmental or insurance bodies), but were more often internally motivated, fueled by the conviction among healthcare professionals that collaboration is key to increase the quality of care. Networks strive to facilitate access to specialized healthcare workers and supportive services, increase the expertise for specific conditions, reduce unwanted variations in care practice, and smoothen care coordination. Optimal collaboration within integrated networks should also boost the experience of care delivery among healthcare professionals. Building such care networks is in line with the World Health Organization (WHO) global strategy on people-centered and integrated health services and their call for “integrated health services that are managed and delivered in a way that ensures people receive a continuum of health promotion, disease prevention, diagnosis, treatment, disease management, rehabilitation and palliative care services, at the different levels and sites of care within the health system, and according to their needs throughout their life course.” (1).

A challenge is that the various existing care networks vary greatly with regard to scale (from local to for example European), focus (single disorder vs. a group of related disorders), extent of care delivery (some are monodisciplinary, others multidisciplinary, but with a great variety of disciplines involved), scope, sustainability, level of professionalism, governance structure, etc. Such variability is explained by many factors. These include financial and infrastructural resources, density of available experts, frequency of the disease(s) covered, density of the overall population, specific characteristics of regional or national healthcare systems, and cultural aspects.

It appears that new initiatives for care networks, well-intended as they may be, are often either re-inventions of the wheel, neglecting the opportunity to learn from previous and current care networks, or duplications of other networks, insufficiently addressing the question of compatibility with specific aims and requirements. We here share our view on several *generic* principles and ingredients for care networks, which can and need to be tuned toward the specific network that is being designed and built. Our view is based on our rich experience with the Dutch ParkinsonNet approach, which we will introduce first. We will then discuss a framework of generic aims and strategies of care networks, as well as the specific challenges related to networks that target rare (movement) disorders.

## ParkinsonNet—History, Merits, and Lessons Learned

The Dutch ParkinsonNet is a multidisciplinary professional network that aims to improve the quality of care delivery for patients with Parkinson's disease (PD) or a form of atypical parkinsonism (2, 3). The network consists of a limited number of specifically trained healthcare professionals (every participant has received an intensive 3-day baseline training course according to the latest guidelines), who attract a high caseload and thereby continuously improve their Parkinson-specific expertise. The network started in 2004 in the Netherlands, motivated by two concurrent developments. The first motivations came directly from clinical practice, where there was a widely felt need for an easily accessible community-based network of allied health professionals with dedicated expertise in treating patients with PD (4). This disorder is characterized by a wide range of motor and non-motor symptoms, many of which respond insufficiently to symptomatic pharmacotherapy (5). Allied health interventions such as physiotherapy, occupational therapy or speech-language therapy can potentially treat many of these otherwise treatment-resistant symptoms, in particular in light of the underlying pathophysiology: basal ganglia dysfunction in PD leads to loss of automated movements, but this can be bypassed using a range of compensatory strategies, such as cueing strategies to improve gait, or specific strategies to improve the intelligibly of speech (6). However, optimal delivery of such interventions requires a good understanding of both the complex clinical presentation and underlying pathophysiology of PD, as well as knowledge of specific treatment strategies. At the time, it was impossible to initiate a dedicated referral to a motivated allied health professional who sufficiently understood PD and who had considerable experience in treating PD patients. Some professionals have built up rich expertise in their daily practice, but they are not readily retrievable in the “yellow pages” of PD. And even when such professionals can be found, it was very difficult to initiate an integrated and multidisciplinary treatment for patients, because most professionals knew very little about what other professional disciplines had to offer (7), and easy communication channels were lacking. A network approach was felt to be an appropriate solution for these challenges.

The second motivation was the need to build a better evidence-base for the various allied health interventions in the field of PD. Although allied health interventions were widely considered to be potentially useful therapies for PD, robust scientific evidence to support the merits of these approaches was lacking. Performing clinical trials was deemed to be a risky enterprise, because at the time, allied health professionals had received very little Parkinson-specific training as part of their routine educational programs, and also treated very few patients in their daily practice annually. Having such poorly experienced providers as the deliverers of care in an intervention trial carried an enormous risk of creating a false-negative result. This further motivated the installation of a network of initially only specifically trained physiotherapists, who were trained according to a newly developed practice-based guideline. This created the necessary infrastructure for subsequent clinical trials.

The first ParkinsonNet network was launched in the eastern part of the Netherlands, and consisted of a small and selected group of healthcare professionals from three different professional disciplines (physiotherapy, occupational therapy, speech-language therapy) (8). All participants were trained according to practice-based evidence guidelines, and were also educated about the offerings of the other professional disciplines. Using simple brochures, referring physicians were informed about the presence of these specifically trained professionals, allowing a dedicated referral and a subsequent increase in caseload. Following positive experiences with this initial small regional network (8), eight further networks were launched in different regions of the Netherlands, consisting initially of only specifically trained physiotherapists, with the aim of using this infrastructure for a subsequent cluster-controlled trial (9). Eight other comparable regions initially served as controls, but following the positive outcome of the trial, these regions also received a professional physiotherapy network. In subsequent years, the network was extended both geographically (reaching a full nationwide coverage by the year 2010), and also in terms of numbers of attached professional disciplines. The network currently exists of over 3,400 specifically trained healthcare professionals, from now 19 different professional disciplines (not only allied health, but also dieticians, Parkinson nurses, social workers, etc.). The active “ingredients” of the ParkinsonNet approach are summarized in Table 1.

**Table 1:** Overview of the supportive building blocks of care networks, including a detailed description and examples of how these have been implemented in ParkinsonNet.

**Building blocks**	**Description**	**The way ParkinsonNet implemented this**
Selection and certification	A selection process combined with a baseline training leads to selective inclusion of motivated and specifically trained healthcare providers. Periodic re-certification based on quality criteria is important to guarantee a high-quality expert network.	• Each year, ParkinsonNet includes new healthcare professionals in the network. In each region we strive to reach an appropriate number of allied health professionals. The required number depends on the discipline and the geographic area. • ParkinsonNet requires members to treat a minimum number of PD patients each year. • Members commit to work according to evidence-based guidelines. • Every 2 years a mandatory re-certification is required based on quality-of-care criteria.
Support centre	An overarching support centre that supports regional networks of providers to work together to improve regional healthcare delivery.	• A national ParkinsonNet coordination centre that provides active guidance to 71 ParkinsonNet regional networks and >3,400 ParkinsonNet healthcare professionals. ◦ Supporting regional networks with personal advice ◦ Financial support to organize regional meetings ◦ Sharing best practices among regional networks ◦ Sharing successful formats for organizing interesting regional meetings
Guideline development and implementation	Guideline development is a solid base for healthcare improvement, both to improve quality of care in daily practice and as important basic training material. Guideline development alone is not enough. It is crucial to support healthcare professionals to work in accordance with guidelines. Accessible guidelines -possibly with some decision support – are a prerequisite for achieving this.	Together with several associations for healthcare professionals, and with the Parkinson Patient Association, ParkinsonNet has developed guidelines: • Monodisciplinary guidelines for physiotherapy, speech-language therapy, occupational therapy, dietary issues, Parkinson's disease nurses and palliative care. • A multidisciplinary guideline, including a consensus-based model for regional and transmural organization of multidisciplinary care
Continuous mono- and interdisciplinary learning	To become an ‘expert’, healthcare professionals should participate in continuous learning cycles, including interaction and information exchange between providers.	• Eligible members must follow a baseline PD-specific training according to evidence-based guidelines (3 days). • After completing this training it is crucial to start learning on the job; expertise can only be increased by treating many patients and by discussing the treatment of complex patients with other health care professionals • Multiple specific trainings, for example about cognition and palliative care • Multiple short animated videos about topics as 10 tips for carers and psychosocial care • Annual conferences • Regional interdisciplinary meetings (twice a year) • Participation in web-based national and regional online communities
Online and offline meetings	Regularly meeting other professionals is important to learn from each other, to inspire each other and to facilitate contact between professionals when this is needed to discuss the treatment strategy for individual patients within a multidisciplinary team.	• Network participants must meet each other in their own region at least twice a year • National annual conference (to learn and meet other professionals) • Online interaction between professionals via an online community tool (ParkinsonConnect)
Visibility and accessibility of experts	Patients should be able to readily find and access specialized experts.	• Providing a web-based search engine (www.ParkinsonZorgzoeker.nl) • Providing experts with a dedicated promotion package to enhance their visibility
Patient education and engagement	Key to patient empowerment is education of patients and the behavior of providers within the individual patient-provider relationship.	• National level: ◦ Strategic partnership with Dutch Parkinson Patient Association ◦ Advisory patient panel for novel technologies or other innovations ◦ Patient representation at regional and national conferences ◦ Educational web-based television program (www.parkinsonTV.nl) ◦ PD management guideline in lay language for patients ◦ Website with reliable information about ParkinsonNet (www.ParkinsonNet.nl) • Regional level: ◦ Collaboration with local branches of Parkinson Patient Association • Individual patient-provider relationship: ◦ Training participating providers to engage patients as partners in healthcare ◦ Promoting shared decision making
Continuous evaluation and improvement of the network	Continuous evaluation and improvement of the network is key. Transparency about performance indicators is also important to inform stakeholders about the merits of the network.	Insight into: • Quality (e.g. adherence to guidelines) • Outcomes (e.g. complications) • Costs • Average caseloads of network participants • Utilization of the network by patients • Experiences of healthcare professionals • Experiences of patients Health care insurance data and surveys among network participants are used to collect this information. Results are published on the ParkinsonNet website.
Supportive technology	Technology can support interdisciplinary collaboration within the network of each individual patient, and also within regional or national networks of health care professionals.	• ParkinsonConnect, a web-based community tool that enables easy communication between healthcare professionals. • ParkinsonTV • ParkinsonNEXT • ParkinsonNet.nl

The merits of this network approach have subsequently been evaluated in a series of clinical studies, including both carefully controlled trials (9–12) and large-scale uncontrolled analyses of a national medical claims database (13). Taken together, these studies provided consistent and converging evidence that supports the cost-effectiveness of a network approach, which appears to be mediated by an improved care delivery (3): the knowledge and use of professional guidelines has enhanced; the caseload of the network participants has increased significantly, not only initially, but the concentration of care continues to improve over the years (14); professionals are much better aware of what other disciplines in the network potentially have to offer; interdisciplinary collaboration has improved; health outcomes are better for patients treated within the network, including a marked reduction in hip fractures and hospital admissions for orthopedic injuries or aspiration pneumonia; and healthcare costs have reduced significantly, as a result of both prevented disease complications and a greater efficiency of care (ParkinsonNet professionals require significantly fewer treatment sessions to achieve their treatment goals). One study even showed a tendency toward a lower mortality rate for patients receiving network care, presumably because of the prevented disease complications (13).

The scientific publications that documented these positive outcomes stirred a fast rising international interest in building similar networks for Parkinson patients in other countries. We have meanwhile introduced comparable networks in, among others, United States (a partnership with Kaiser Permanente in California, and a network in Rochester), Norway and Luxembourg, while additional trainings are currently taking place or are being planned in Germany, Italy and China. An important lesson learned from this international experience is that the networks in other countries each time have to be adjusted to the local needs, as well as to the existing infrastructure and available services. Supporting other countries in building similar networks was all but a “copy paste” enterprise, but instead was always preceded by a careful inventory of what existing services were already operating well, which challenges existed regionally, and which of the solutions offered by ParkinsonNet could help to address these existing challenges. A further important lesson was that each international network is to be governed by regional leadership, and that it is essential to locally train “super experts” for each associated professional discipline, so these can subsequently oversee the quality of the regional network in the other country. Capitalizing on these lessons, the initial experience with the international network in California has been very positive, showing a significant change in referral patterns toward the specifically trained ParkinsonNet participants (15).

We previously already alluded to the opportunity to consider ParkinsonNet as a scalable model for other chronic conditions, including other movement disorders (3). The management of these disorders is equally challenged by very comparable issues such as lack of specific expertise, care fragmentation, and insufficient collaboration between disciplines. These challenges are presumably even more prominent for rare disorders, for which dedicated expertise and optimal collaboration with fellow peers in the expert network is presumably extra important. Most of the key components of ParkinsonNet can be considered as generic ingredients (“building blocks”) for care networks, although the specific requirements for each professional network will undoubtedly have to be adjusted to the unique needs of each condition as well as the specific regional circumstances. Next, we will discuss these generic ingredients, which we refer to as supportive building blocks, according to the why, how and what principles.

## Supportive Building Blocks of Care Networks

### Why?

**Figure 1:**
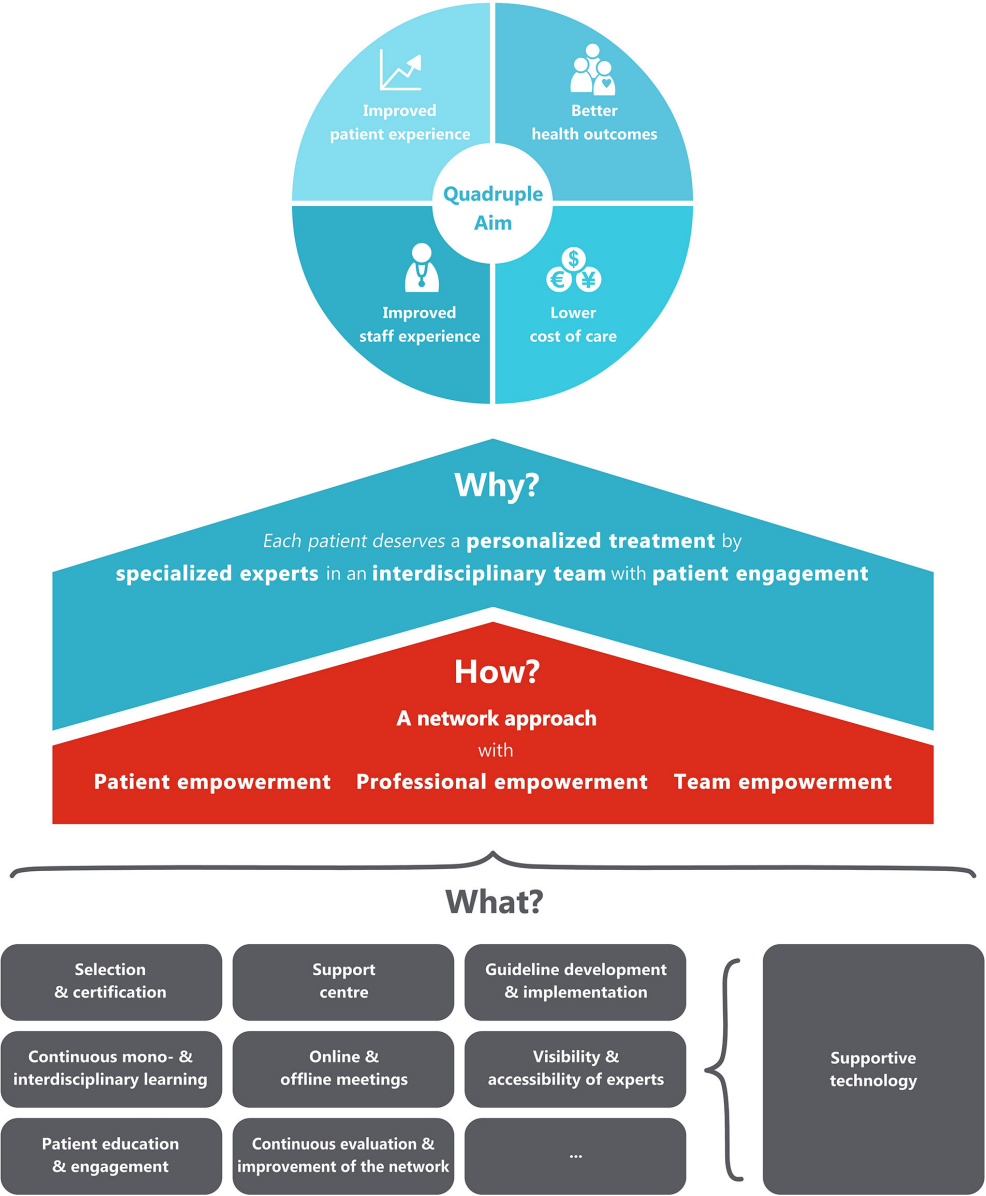
Proposed universal model for integrated network care.

The ultimate objective of the integrated network care model is to reach the quadruple aim of healthcare, with (1) better health outcomes, (2) lower cost of care, (3) improved patient experience, and (4) improved staff experience (Figure 1). To reach this quadruple aim, the model focuses on four concrete goals: (1) patients should always receive personalized treatments, (2) care is delivered by professionals with adequate specific expertise for the disease at hand (specialized experts), (3) experts from different disciplines and organizations should work together with other professionals within interdisciplinary teams (interdisciplinary care), and (4) patients should be seen as real partners in the healthcare process, and be supported to make an active contribution to their own health (patient engagement). These driving forces are in line with the values of integrated care identified by Zonneveld et al. (16) in their systematic review that aimed to identify factors that drive behavior, decision-making, collaboration and governance processes within integrated care networks.

### How?

Central to the model that we present here is a managed multi-level network. In this network approach, patients and professionals from different disciplines and organizations work closely together in different networks and at different levels, in order to improve the quality of care for all patients with the same disease in a certain geographical area. In this regard, it is good to realize that the word “network” is often used to denote various forms of collaboration at very different scales. The smallest scale of a network is formed by the healthcare team that is involved in the management of any given individual patient. To provide a concrete example, the neurologist, nurse, physiotherapist, dietician and general practitioner together from the personal care network of Mr. Johnson. But networks also exist at a larger scale. Specifically, at a higher level, larger numbers of healthcare professionals, all with dedicated expertise in the same disease, can meet each other in local, regional or national networks, not so much to provide care to an individual patient, but rather aiming to learn from each other and to agree about regional healthcare management issues (for example, do we have a sufficient number of specifically trained physiotherapists to optimally manage the population within this specific geographical area?). The geography of these networks depends on the incidence of a disease. For diseases with a high prevalence, regional or even local networks of professionals can be formed. For diseases with a much lower incidence, these networks can be organized at a state or national level. The members of the regional networks should ideally be supported by a national support center, which provides among others generic support and advice to the various regions. Patients, professionals and teams are empowered by this support center with a range of activities to reach the three goals. The most important activities are described as the building blocks in the “what-section.” In the building process of the care network, four development phases of integrated care can be followed: “initiative and design phase,” “experimental and execution phase,” “expansion and monitoring phase,” and “consolidation phase.” These phases have been described and validated by Minkman et al. (17, 18).

### What?

The generic building blocks to organize the network and to empower patients, professionals and teams are presented in Table 1. This overview of building blocks is not inclusive, and the building blocks can differ between diseases, and will also depend on the specific regional circumstances, including the characteristics of the healthcare system. For example, in the aforementioned collaboration between the Dutch ParkinsonNet and Kaiser Permanente in California, a decision was reached to use only a restricted number of building blocks (e.g., guideline development, professional training, patient empowerment), whereas others were deemed to be unnecessary (e.g., use of some of the supporting digital technologies, since these were already available as part of the Kaiser Permanente offerings) (15).

## Specific Ingredients and Challenges for Rare Movement Disorders

The main challenges for networks that focus on rare disorders are obviously related to the rarity of each of the individual conditions. There are estimated to be 600–800 different rare diseases, each with individually low prevalence rates. We should therefore not strive to have specifically dedicated networks for each of the rare movement disorders separately, also because there are often mixed types of movement disorders in these conditions (for example, many patients with a hereditary ataxia may present with additional movement abnormalities, such as dystonia or chorea).

As expertise results not only from having received a baseline training but also to a large extent from accumulating experience in daily clinical practice, a sufficient exposure to large numbers of patients with a certain condition is required. This clearly necessitates efforts to centralize the care for specific conditions among a restricted number of specifically trained professionals. The concentration of care was a successful cornerstone of the ParkinsonNet approach, but this will presumably be at least as important for networks focusing on rare movement disorders. In this regard, it is a promising development to see the presence of an increasing number of expert centers for rare disorders (sometimes self-proclaimed, but increasingly also formally recognized according to established objective criteria) in many countries. In accordance with our ideas about the supportive building blocks (Table 1), such experts centers would be ideally positioned to take on the role of support centers when new networks are being formed.

To be designated as an expert center by national authorities requires that various predefined criteria have to be met, which serve a quality control purpose. These criteria should address relevant issues such as minimal patient numbers, optimal team size and composition, research performance, and other tangible metrics. A next step would be to evaluate these centers based on actual patient-relevant performance measures, but such quality-of-care criteria remain to be established for rare movement disorders.

In the Netherlands, we have seen a steep rise in the number of expert centers, sometimes even for a single rare disease. As a response, future applications should target clusters of rare diseases in order to obtain a formal recognition by the Ministry of Health. Such clustering can be reached at various levels, such as a comparable etiology, overlapping functional deficits, or similarities in treatment. This clustering also serves to achieve a certain caseload, which is needed to develop, maintain and ultimately expand the required level of expertise. Clustering could lead to a dilution of expertise for single disease entities, but the advantages and necessity hereof outweigh this potential disadvantage. Moreover, one could argue that recognizing the overlap between and co-existence of multiple movement disorders—which is more likely secured in centers that host clusters—actually aids the disease-specific expertise.

One example of how clusters for rare movement disorders could look like, are those that have been proposed within the European Reference Network for Rare Neurological Diseases (ERN-RND; see contribution by (19); doi: 10.3389/fneur.2020.616569). This network has a strong focus on rare movement disorders, and has used (1) cerebellar ataxias and hereditary spastic paraplegias, (2) Huntington's disease and other choreas, (3) dystonia, paroxysmal disorders, and neurodegeneration with brain iron accumulation, (4) atypical parkinsonian syndromes as the four main clusters.

Ideally, all patients with rare movement disorders should be seen, at least once, in expert centers, in particular to establish a definitive diagnosis whenever possible, and to outline the contours of a therapeutic management program for the following years. However, this will not always be possible, for example because of long travel distance and costs or patient immobility, or simply because of insufficient capacity. Additionally, lack of awareness of the presence of centers of expertise further hampers a dedicated referral to these centers. Realizing that physical consultations are not always feasible, we feel that an important task of such an expert center within the network structure is to transfer knowledge and skills to local healthcare professionals working close to the patients' home. Many patients prefer to be (also) followed up by their local neurologist, and from studies in the PD field we now know that neurologists who work in community hospitals deliver better quality care for patients if they are supported remotely by an expert via telemedicine (so-called peer-to-peer consultations) (20).

One of the main challenges in the allied health domain is to identify professionals who are indeed motivated to be equipped with greater knowledge and better skills for a specific rare disease, of which the total number of patients in their practices will remain extremely low. This is in clear contrast with the original ParkinsonNet model, where trained participants in the network have witnessed a very tangible increase in the number of patients with PD in their daily practice. This notion raises the question where for example allied healthcare interventions should be delivered best. This could still a local trained therapist, but may also very well be a rehabilitation facility as close as possible to the patient. Regardless of the scenario, well-designed and preferably evidence-based guidelines are needed. For rare movement disorders, such guidelines are often lacking, particularly for non-pharmacological interventions. The absence of such guidelines—a crucial building block in the model we present here—makes it very difficult to have local professionals execute an intervention proposed by an expert center. Having guidelines is also essential as baseline training materials for professionals who wish to join a professional network, and to help reduce unwanted variations in care delivery. For some of these guidelines, particularly those that involve rehabilitation, one will need to consider clustering at the level of shared or overlapping movement disorders, as eluded to earlier. This has also been done in ParkinsonNet, where professionals now deliver care to both PD and atypical parkinsonism patients. A treatment guideline for ataxia will benefit patients with MSA-c and Freidreich's ataxia alike, while separate guidelines will prevent professionals to become acquainted with the commonalities in symptoms, functional deficits, and treatment principles. Some recent studies on rehabilitation in for example ataxia and cervical dystonia will be useful starting points for the development of such guidelines, which should have priority for rare movement disorders (21, 22).

The quality of care provided by expert (and support) centers will be improved further if there is between-center collaboration and knowledge exchange. To achieve this, cross-state and international networks of expert centers have been established, e.g., the European Reference Networks (ERN). By demanding clustering, the European Union wisely prevented the emergence of too many networks that deal with a single or limited set of diseases. One of the ERN's specifically addresses the cluster “rare neurological disorders” (ERN-RND; see contribution by Reinard et al. in this series), including movement disorders. This development offers opportunities to widely harmonize disease management, to deliver cross-border care, to provide access to facilities to low-resource countries, and to draft joint research programs. While advantages are omnipresent, such international networks do, however, also add layers of complexity, such as reimbursement issues for cross-border care, complex network governance, and asymmetry in knowledge and resources that prevent guideline harmonization. An elegant solution to provide cross-border care, or at least get access to an international panel of experts, is provided by an IT-platform that the European Union has installed for ERN's, in harmony with “supportive technology” identified by us as one of the building blocks.

## Concluding Remarks

Based on our experience with ParkinsonNet, a network for integrated care in PD, we have here shared our view on a universal model for care networks. We have presented supportive building blocks of such a network, which are generic ingredients that ultimately allow the network to reach the quadruple aim of healthcare. The rarity of various movement disorders imposes certain unique challenges and barriers that prevent a full and immediate adoption of the model as laid down here. However, our view on the generic ingredients can serve as the starting point for shaping a new care network. Also, existing networks and centers that are part of these networks can identify which building block(s) they wish to adopt or improve, which can be jointly and transparently prioritized. Ideally, innovations such as care networks should be tested against current standards of care and demonstrate added benefit and/or cost-effectiveness. We appreciate that this will be a challenge on its own for cross-border networks for rare disorders.

For rare movement disorders, it seems that three aspects have priority. First, clustering of rare movement disorders at the network level is needed, not only to identify the expert professionals and centers, but also to ensure a certain caseload and to exploit the fact that specific care interventions may overlap across different conditions. Second, supportive technology is a true necessity in order to facilitate exchange of and access to knowledge and expertise. Technological solutions are particularly important, because the density of experts and expert centers for rare movement disorders is low in most regions and countries, so physical in-person meetings are difficult to organize. In fact, the many challenges imposed by the unfolding COVID-19 crisis have only further helped to accelerate the introduction of telemedicine solutions to ascertain a good quality of care delivery for people living with chronic neurological conditions (23). Lastly, interdisciplinary guidelines need to be developed, as these are quite central to the model, facilitating training of professionals, harmonizing care, and evaluating performance of professionals, centers and the network.

## Data Availability Statement

The original contributions presented in the study are included in the article/supplementary material, further inquiries can be directed to the corresponding author/s.

## Author Contributions

All authors listed have made a substantial, direct and intellectual contribution to the work, and approved it for publication.

## Conflict of Interest

The authors declare that the research was conducted in the absence of any commercial or financial relationships that could be construed as a potential conflict of interest.
